# Hypoxia Imaging and Adaptive Radiotherapy: A State-of-the-Art Approach in the Management of Glioma

**DOI:** 10.3389/fmed.2019.00117

**Published:** 2019-06-12

**Authors:** Michael Gérard, Aurélien Corroyer-Dulmont, Paul Lesueur, Solène Collet, Michel Chérel, Mickael Bourgeois, Dinu Stefan, Elaine Johanna Limkin, Cécile Perrio, Jean-Sébastien Guillamo, Bernard Dubray, Myriam Bernaudin, Juliette Thariat, Samuel Valable

**Affiliations:** ^1^Normandie Université, UNICAEN, CEA, CNRS, ISTCT/CERVOxy Group, GIP Cyceron, Caen, France; ^2^Department of Radiation Oncology, Centre Lutte Contre le Cancer François Baclesse, Caen, France; ^3^Department of Radiophysics, Centre Lutte Contre le Cancer François Baclesse, Caen, France; ^4^Team 13–Nuclear Oncology, INSERM U1232 Centre de Recherche en Cancérologie et Immunologie Nantes Angers (CRCINA), Nantes, France; ^5^Department of Radiotherapy, Gustave Roussy, Université Paris-Saclay, Villejuif, France; ^6^Normandie Université, UNICAEN, CEA, CNRS, ISTCT/LDM-TEP Group, GIP Cyceron, Caen, France; ^7^Department of Neurology, Centre Hospitalier Universitaire de Nîmes, Nîmes, France; ^8^Département de Radiothérapie et de Physique Médicale, Laboratoire QuantIF–LITIS [EA 4108], Centre de Lutte Contre le Cancer Henri Becquerel, Université de Normandie, Rouen, France

**Keywords:** glioblastoma, hypoxia, imaging, PET, MRI, radiation therapy

## Abstract

Severe hypoxia [oxygen partial pressure (pO_2_) below 5–10 mmHg] is more frequent in glioblastoma multiforme (GBM) compared to lower-grade gliomas. Seminal studies in the 1950s demonstrated that hypoxia was associated with increased resistance to low–linear energy transfer (LET) ionizing radiation. In experimental conditions, the total radiation dose has to be multiplied by a factor of 3 to achieve the same cell lethality in anoxic situations. The presence of hypoxia in human tumors is assumed to contribute to treatment failures after radiotherapy (RT) in cancer patients. Therefore, a logical way to overcome hypoxia-induced radioresistance would be to deliver substantially higher doses of RT in hypoxic volumes delineated on pre-treatment imaging as biological target volumes (BTVs). Such an approach faces various fundamental, technical, and clinical challenges. The present review addresses several technical points related to the delineation of hypoxic zones, which include: spatial accuracy, quantitative vs. relative threshold, variations of hypoxia levels during RT, and availability of hypoxia tracers. The feasibility of hypoxia imaging as an assessment tool for early tumor response to RT and for predicting long-term outcomes is discussed. Hypoxia imaging for RT dose painting is likewise examined. As for the radiation oncologist's point of view, hypoxia maps should be converted into dose-distribution objectives for RT planning. Taking into account the physics and the radiobiology of various irradiation beams, preliminary *in silico* studies are required to investigate the feasibility of dose escalation in terms of normal tissue tolerance before clinical trials are undertaken.

## Introduction

### Brain Tumors and Hypoxia

Brain tissue physiologically has a tissue pO_2_ (ptO_2_) of ~40 mmHg, referred to as a normoxic or aerobic state. Hypoxia, generally defined when ptO_2_ falls below 10 mmHg, is the result of an imbalance between oxygen consumption and delivery, a common situation in various types of malignancies.

Tumor growth was initially modelized by Gompertzian curves in the 1970s, in which the growth saturates when the tumor volume reaches the carrying capacity (1, 2). However, this model has some limitations and has been improved by incorporating various parameters such as angiogenesis and necrosis. A specific focus was placed on hypoxia, known to play a crucial role in tumor angiogenesis, genetic instability, and tumor invasion (3). More recently, hypoxia has also been shown to induce pro-tumoral activity by macrophage polarization (4). It is evident that hypoxia has a positive role in tumor growth and a negative role in therapeutic response (5) and is ultimately related to poor prognosis (6–8).

In primary brain tumors, hypoxia is also associated with malignant tumor growth. Glioblastoma multiforme (GBM), the most aggressive glioma and most frequent primary brain tumor, is particularly hypoxic. Using the Eppendorf needle electrode, previous works demonstrated that while the oxygenation in the normal brain ranges around 40 mmHg of oxygen, it falls below 10 mmHg in GBM (9, 10). However, hypoxic components are highly heterogeneous both within a single tumor and among patients. It has been proposed that tumors could be separated into three compartments: well oxygenated, acutely hypoxic, and chronically hypoxic (11).

Hypoxia also induces resistance to radiotherapy (RT) (12). In the early 1950s, Gray and colleagues reported that the radiosensitivity of mammalian cells was dependent on oxygen concentration (13). Hypoxia was therefore assumed to contribute to the failures after RT in cancer patients. It has also been suspected to be involved in resistance to various chemotherapies (14, 15). Explored solutions to target hypoxia included the use of hyperbaric oxygen chambers, hypoxic radiosensitizers, and, in recent years, hypoxia image guided radiotherapy (HIGRT) (16).

More recently, various publications have demonstrated that hypoxia changes during tumor growth. Hypoxia is a result of an increased oxygen demand not only from tumor cells but also from immune cells, coupled with a perturbed vasculature (17). While in normal situations, the capillary density allows oxygen to be delivered to the cells with distances ranging from 30 to 60 μm, within a tumor, the distance to the closest capillary dramatically increases and causes a decrease in oxygen pressure. The concept of perfusion-limited hypoxia resulting from vessel obstruction and perturbed blood flow (poorly oxygenated blood) has introduced the concept of dynamic or cycling hypoxia (18–20). Temporal instability of ptO_2_ has been observed with intermittent periods of reoxygenation. The kinetics of cycling hypoxia follow a complex timescale and occur with two frequencies: a few cycles per hour and cycles lasting from hours to days (21, 22). At present, no clear distinction exists between chronic and cycling hypoxia.

### Hypoxia and Radiobiological Basis

In the presence of molecular oxygen at the time of or within microseconds after exposure, low-LET radiation ionizes water molecules, producing high-energy electrons and highly reactive oxygen species (ROS) (23). DNA damage results from either a direct or an indirect (via ROS) effect of irradiation. In the absence of oxygen, ROS are not produced, and DNA damage is reduced for a given RT dose. *In vitro*, the ratio of the doses yielding the same level of cell mortality in anoxic (100% N_2_ atmosphere) vs. oxic (100% O_2_ atmosphere) conditions is 2.5–3, corresponding to the oxygen enhancement ratio (OER) (24–26). This “oxygen effect” is not associated with oxygen-dependent differences in DNA repair processes (27). Therefore, oxygen is considered as the strongest existing radiosensitizing agent. Hypoxic tumors are thus considered radioresistant and are harder to control with conventional RT doses.

**Figure 1:**
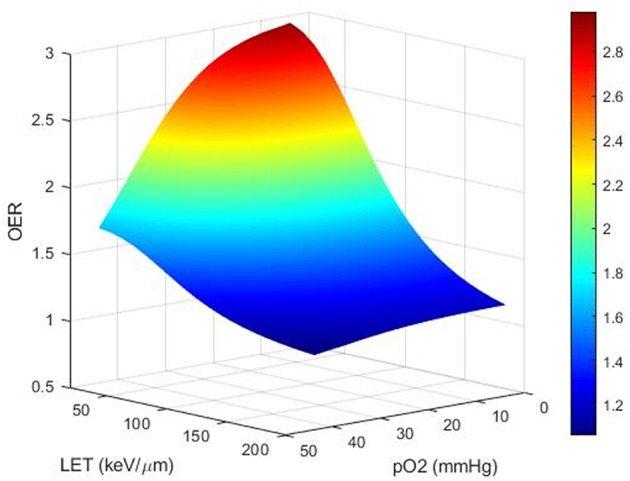
Theoretical computational modeling of the OER as a function of pO_2_ and LET (performed on MATLAB). OER increases nonlinearly with increasing degree of hypoxia and decreases with increasing LET. Compared to low-LET conventional RT (photons or protons), high-LET RT, over a few hundreds of keV/μm (carbons), is expected to be less sensitive to hypoxia and could be more efficient for treating hypoxic tumors.

OER and OER modeling: As a function of pO_2_ and LET, OER increases nonlinearly with decreasing pO_2_ as described by the Alper and Howard-Flanders formula (28) and with decreasing LET (27–29) (Figure 1). Under exposure to low-LET radiation, OER is around 2 for a pO_2_ value of around 10–15 mmHg, and a maximum is reached with pO_2_ <5 mmHg (30, 31). For high LET (over a few hundred keV/μm), OER remains around 1, whatever the pO_2_ (29, 32). Thus, high-LET radiation therapy is supposed to be more efficient than low-LET conventional RT (photons or protons) when treating hypoxic tumors (33, 34). This could be explained by the *in situ* “oxygen production in the heavy ion track” phenomenon (35–38).

For a precise modeling of OER dependence, a rigorous analysis should include several parameters: ptO_2_ in both the hypoxic and aerobic conditions, LET, cell survival end point, dose per fraction, particle species, tissue, and cell cycle phase. These variables are derived from *in vitro* survival data and may overestimate or underestimate the effects of hypoxia *in vivo*. Due to the complexity of dependencies, results of experimental data on OER measurements possess significant uncertainty. Improved understanding of the physical and chemical basis of the OER would add useful information on top of current empirical models. An accurate OER model is necessary to calculate doses necessary for RT dose escalation. Numerous mathematical OER models have been proposed, based on a range of experimental data from literature (Figure 1). However, the optimal mathematical function remains unknown, and estimation remains empirical. Once known, the model will be of invaluable aid to radiation oncologists in performing “hypoxia dose painting” in treatment planning for photon and ion beam RT.

Characterizing the heterogeneity of hypoxia necessitates tools with good temporal and spatial resolution to enable its eventual use in personalized medicine. Medical imaging is a promising tool, as it allows repeated noninvasive measurements to track both the temporal and spatial heterogeneity of tumor hypoxia. This is particularly relevant in RT, where constant technological advancements may permit treatment personalization based on the local ptO_2_. There are, however, numerous points that require validation before using imaging of hypoxia for radiation therapy guidance.

## Mapping of Hypoxia in Clinical Situations: Current Developments

Various approaches have been designed to assess hypoxia in tissues. The use of implantable probes or needles is still the gold standard for ptO_2_ measurement (5). In a clinical environment, however, tissue ptO_2_ cannot be mapped with probes (39), and biomedical imaging based on positron-emission tomography (PET) and magnetic resonance imaging (MRI) serves as a surrogate biomarker of hypoxia or of cerebral oxygenation (Table 1).

**Table 1:** Imaging biomarkers to evaluate oxygenation in glioblastoma: advantages and limitations.

	**Advantages**	**Limitations**
StO_2_	•Easy setup and application in clinical routine •Sensitive •Assuming fully oxygenated arterial blood, the fraction of deoxygenated blood corresponds to the OEF •Spatial resolution is better than PET biomarkers	•Indirect assessment of p_t_O_2_ •Specificity for hypoxia needs to be validated
OE-MRI	•Showed promising results in the characterization of intratumor hypoxia heterogeneity in one GBM model •Spatial resolution is better than PET biomarkers	•Indirect assessment of p_t_O_2_ •Specificity for hypoxia needs to be validated •Needs to be validated in other GBM models and in the clinical setting
MOBILE	•No need to inject contrast agent •Spatial resolution better than PET biomarkers	•Indirect and relative assessment of p_t_O_2_ •No studies in brain tumors
MR fingerprint	•Multi-parametric (vascularization, oxygenation…) characterization with rapid acquisition •Spatial resolution is better than PET biomarkers	•Indirect and relative assessment of p_t_O_2_ •Needs to be validated in other GBM models and in the clinical setting with multiple slices
^15^O-oxygen	∙Allows direct quantification of OEF	•Very short radioactive decay •No linear relation between oxygen consumption and cellular hypoxia •Spatial resolution
[^18^F]-FMISO	•Current gold standard for hypoxia imaging •Indicator of cellular hypoxia	•Injection of a radioactive compound •Relatively prolonged time before steady-state acquisition (2h) •Spatial resolution
[^18^F]-FAZA	•Indicator of cellular hypoxia •More rapid clearance than [^18^F]-FMISO	•Injection of a radioactive compound •Needs to be validated in a more important number of studies •Spatial resolution
[^18^F]-HX4	•Indicator of cellular hypoxia •More hydrophilic tracer allowing more rapid clearance than [^18^F]-FMISO/FAZA	•Injection of a radioactive compound •Not recommended for brain tumors •Spatial resolution
[^18^F]-DiFA	•Indicator of cellular hypoxia •More hydrophilic tracer allowing more rapid clearance than [^18^F]-FMISO/FAZA	•Injection of a radioactive contrast agent •Needs to be validated in a more important number of studies •Spatial resolution
[^62^Cu]/[^64^Cu]-ATSM	•Characterization of moderate hypoxia •Promising tracer for imaging hypoxia thanks to its high membrane permeability and low redox potential	•Injection of a radioactive compound with long half-life (12.7 h) •Specificity to hypoxia is questionable •Spatial resolution

### MRI Markers

MRI has the advantage of being nonionizing and can be used to quantify the blood oxygenation level in tissue (StO_2_) (40). In particular, a BOLD-based MRI method for the measurement of relative oxygen extraction fraction (rOEF) showed that high rOEF was present in high-grade but not low-grade gliomas. However, confounding factors such as cerebral blood volume (CBV), tissular T2, and contrast agent leakage need further investigation (41). Oxygen-enhanced MRI (OE-MRI) is likewise useful, based on the correlation between hypoxia and the variation in longitudinal relaxation rate (ΔR1) during oxygen challenge (42). In a preclinical model of GBM, Fan et al. have shown that OE-MRI is able to show intratumoral hypoxic heterogeneity and present an interesting correlation of OE-MRI with hypoxia by histological staining (24). However, OE-MRI still has to be validated in other GBM models and in the clinical setting. Mapping of oxygen by imaging lipids relaxation enhancement (MOBILE) (25) has also been proposed and also needs validation. OE-MRI and MOBILE present the advantage of repeated measurements of oxygenation without the need for exogenous contrast agents. Recently, an original approach termed MR fingerprint has also been proposed, which simultaneously obtains data on CBV, mean vessel radius, and blood oxygen saturation and creates high-resolution parametric maps of the microvascular network of the brain (26).

### PET Markers

PET can also be used to map the OEF with radioactive molecular oxygen (^15^O_2_) as a tracer. It can also be used to estimate ptO_2_ by mapping of tracers trapped in areas with low ptO_2_. This approach is achieved with a variety of PET tracers based on an imidazole structure such as 3-[^18^F]fluoro-1-(2-nitro-1-imidazolyl)-2-propanol ([^18^F]-FMISO) (43, 44) and [^18^F]-fluoroazomycin arabinoside ([^18^F]-FAZA), the uptake of which depends on a ptO_2_ threshold (45). After cell penetration by passive diffusion, these tracers are reduced in a two-step process, with the first step being reversed if oxygen is present and with the tracer becoming irreversibly trapped in the absence of oxygen.

It takes time to visualize hypoxic regions using [^18^F]-FMISO or [^18^F]-FAZA due to lipophilicity and slow clearance in normoxic tissues. More recently, 3-[^18^F]fluoro-2-(4-((2-nitro-1H-imidazol-1-yl)methyl)-1H-1,2,3-triazol-1-yl)propan-1-ol ([^18^F]-HX4 or 18F-flortanidazole) (46) and 1-(2,2-dihydroxymethyl-3-[^18^F]fluoropropyl)-2-nitroimidazole ([^18^F]-DiFA) (47) have been developed as more hydrophilic tracers with the potential advantages of shorter acquisition times. However, formal validation in clinical situations is required.

**Figure 2:**
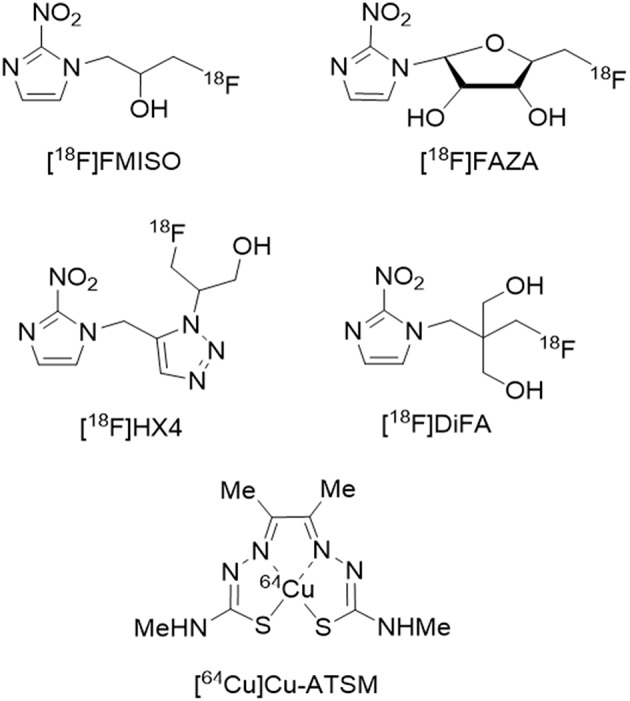
Chemical structure of the various PET tracers designed for hypoxia imaging.

Other radiopharmaceuticals have been described. Cu(II)-diacetyl-bis(N4-methylthiosemicarbazone) (^64^Cu-ATSM) seems to be a promising tracer for imaging hypoxia thanks to its high membrane permeability and low redox potential. However, the selectivity of Cu-ATSM to hypoxia has been challenged and discussed (48). See Figure 2 for the chemical structures of the various PET tracers designed for hypoxia imaging.

## Robustness and Accuracy of Available Techniques to Assess Hypoxia in the Brain

MRI and PET biomarkers have the advantage of being available and regularly utilized in the clinics; however, in assessing hypoxia, they have several limitations, which presently hinder routine clinical utilization for RT dose modulation (Table 1).

### Limitation of MRI Markers

Mapping StO_2_ or the OEF yields a continuous signal with high temporal and spatial resolutions, but the relationship to ptO_2_ is indirect, and vascular changes indirectly reflect tissue changes. In particular, their relationship depends on the dissociation curve of hemoglobin, which itself depends on pH and temperature, among other factors. For example, a lower blood pH or a higher blood temperature would lead to a higher blood ptO_2_ for the same blood oxygen saturation.

In addition, OE-MRI and MOBILE have to be validated in various GBM models and in the clinical setting. MR fingerprint has been validated in patients but only for a single slice; thus, further developments are necessary.

### Limitation of PET Markers

Accessibility: One of the main drawbacks of the extensive use of PET tracers of hypoxia in oncology is that tracer production is cost-intensive and only available at selected centers, in part due to limited manufacturers.

#### Poor Spatial Resolution

As discussed in the review of Grimes et al. (49) the molecular effect of oxygen is in the range of nm to μm, while PET resolution is about 3–4 mm. This raises various concerns about the interpretation of the PET results. It was shown that the PET signal would be similar between two voxels if 25% of a voxel was anoxic (but viable) and the remainder well oxygenated, if the voxel was 50%/50% split between 1.4 mmHg and oxic, or if the whole voxel was at 4.2 mmHg (49).

#### Impact of Altered Blood Flow in Tracer Uptake

PET tracers are delivered to the hypoxic tumor cells via the bloodstream. However, GBM vascularization is highly perturbed, which could impact the tracer biodistribution, notably in anoxic areas without any functional vascularization where delivery of the tracer might not be achieved (50). This could result in a very low tracer uptake in highly hypoxic areas. Vessel permeability may also have an impact in tissue biodistribution if more hydrophilic tracers have to be used. Dynamic PET has been proposed as an alternative to address the issues of both tumor perfusion and hypoxia, but the increased duration of the examination is a limitation for its routine use.

#### Poor Temporal Resolution

The 109-min half-life of ^18^F is hardly ideal for examining temporal resolution. In general, the radioactive nature and short half-lives of PET tracers make it difficult to assess the evolution of hypoxia over hours or days. For instance, a study on head and neck cancers demonstrated that variability in spatial uptake can occur between repeated ^18^F-FMISO PET scans (51). These results could be either a reflection of the poor reproducibility of FMISO PET due to confounding influences (perfusion, permeability) or a reflection of cycling hypoxia.

Molecular oxygen, with its very short half-life, would in theory address the dynamic nature of tissue oxygenation. However, its access is limited to a few centers worldwide, and ^15^O has a poor intrinsic spatial resolution in comparison to ^18^F.

In summary, while being of major importance for tumor growth and resistance to treatment, the mapping and routine assessment of hypoxia remains a challenge. Among the various markers, [^18^F]-FMISO PET remains the most extensively studied and most accurate approach to map hypoxia in the clinical situation (52), but for brain tumors where PET imaging is not standard practice, MRI may provide surrogate biomarkers of oxygenation.

## Hypoxia From the Radiation Oncologist's Point of View

### Hypoxia and the Dose Painting Concept

At present, the same radiation dose is delivered to all subregions of the tumor volume regardless of their individual biology and radiosensitivity. The RT concept of dose painting involves adapting the dose prescriptions for tumor subvolumes as a function of the tumor's heterogeneous biology. This could be done with functional imaging that maps different dose–response levels (53) over anatomic contours provided by morphological imagery, resulting in a “biological target volume” (BTV), where dose escalation could be applied. Hypoxia imaging could be used to provide the level of ptO_2_ and, subsequently, the spatial distribution of potentially radioresistant regions (54). These hypoxic target volumes (HTVs) are given a higher dose to achieve better tumor control (54), taking care not to compromise normal tissue tolerance (55, 56). To counteract radioresistance associated with hypoxic tumors, radiation oncologists need accurate calculations of the biologically optimal RT doses. The technical feasibility of optimizing RT plans has been well documented, mostly in head and neck cancers (57, 58). A similar study has never been done in gliomas.

**Figure 3:**
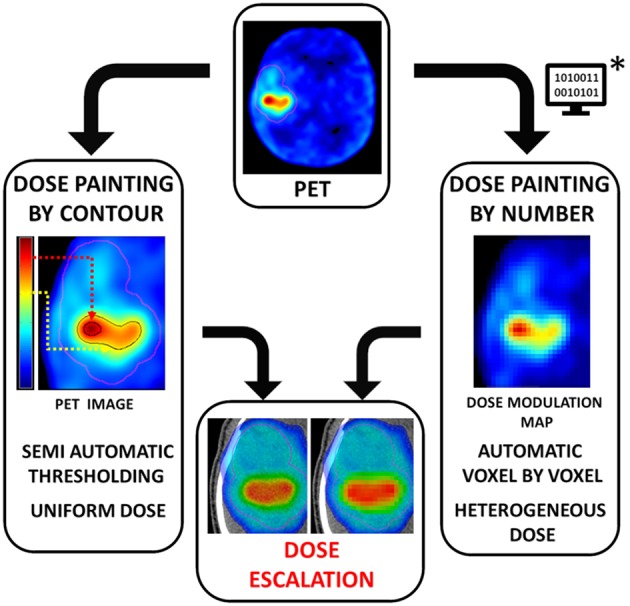
The two main approaches of dose painting: by contour (DPBC) or by number (DPBN). For DPBC, added to the standard clinical dose level (in pink), the radiation oncologist manually delineates a uniform HTV (in black) corresponding to a subjective PET-uptake level threshold (dashed line). Note that both methods use PET images, but DPBN requires a mathematical data pre-processing step (^*^) that computes PET image into a “dose modulation map.” When performed, dose painting allows RT dosimetric simulation for optimal dose escalation.

To define the HTV, there are two main approaches: dose painting by contour (DPBC) or by number (DPBN) based on PET images (Figure 3).

#### Dose Painting by Contour

Also called multilevel or subvolume boosting, DPBC defines the HTV by segmenting a volume based on an uptake threshold on hypoxia functional images. This approach delivers a uniform boost dose to hypoxic subvolumes (59). Pixels with intensities higher than a defined value are considered as potentially hypoxic volumes. The cutoff is based on an empirical uptake threshold relative to a well-oxygenated reference, such as tumor-to-muscle and tumor-to-blood ratios (>95% of normal tissue voxels had a tissue/blood ratio of ≤ 1.2) or SUV>1.4 (60, 61).

DPBC is the most common approach in studies for several reasons. First, it is easier to integrate into conventional clinical workflows using commercial RT treatment planning systems (TPS). Second, it is easier to prescribe uniform dose boost regions. Lastly, it is more robust to spatial errors (62). In practice, dose escalation is achievable for the vast majority of cases (63, 64). However, the absence of consensus on the most appropriate threshold cutoff, the fact that high values can be found outside the tumor, and disparate tracer characteristics (intrinsic biochemical, uptake, clearance, etc.) make this method clinically debatable.

#### Dose Painting by Numbers

DPBN is a voxel-by-voxel level dose prescription based on a relationship between the intensities of neighboring voxels integrated in a “dose modulation map.” This is achieved through a mathematical transformation of the spatial distribution of hypoxia from noninvasive methods such as PET scans, named “ptO_2_map.” The ptO_2_ and OER levels enable algorithms to compute the heterogeneous doses to be prescribed (65, 66).

Several attempts have been made to estimate ptO_2_ and include OER in RT treatment planning. However, these methods are much more complex than DPBC and require specific algorithms and software for numerical processing steps (67). Some methods are proposed (68) but remain subject to discussion (69). Some authors consider a linear transformation of the image intensity into a prescribed dose (65, 67), whereas others assume a “dose redistribution” between hypoxic and normoxic pixels resulting in the same average dose as a conventional RT plan (58, 62, 70).

For head and neck tumors, Toma-Dasu et al. used a nonlinearity approach, which considers that the relationship between [^18^F]-FMISO uptake and ptO_2_ follows a hyperbolic function (65). This equation was adapted for brain tumors and fine-tuned patient by patient using two healthy regions of interest for calibration of the model (68). This approach enables the computation of ptO_2_ maps. However, once ptO2 maps are calculated, dose modulation maps must also be computed. To do this, authors reported an equation that links dose modulation to ptO_2_ by incorporating the OER effect (65). Another approach used was to compute an inverted dose prescription map that can be directly imported into the RT TPS without any modifications (71). To the best of our knowledge, these dose modulation maps have never been proposed for brain tumors.

To conclude, the adaption in clinical practice of both DPBC and DPBN to address tumor hypoxia remains to be validated before becoming a clinical routine.

### Intensity-Modulated Radiation Therapy

In GBM, the standard RT dose prescription is 60 Gray, in 1.8–2 Gray daily fractions, administered 5 days per week for 6 weeks. However, radioresistance is almost constant, inevitably leading to subsequent tumor relapse (72). RT dose escalation is one of the avenues of research being explored to improve local control (73). Because GBMs are infiltrative, diffuse, and often diagnosed late, these usually require irradiation of large volumes encompassing normal brain tissue. Thus, increased doses may potentially lead to unacceptable radiation-induced toxicities (edema, inflammation, necrosis, etc.) and severe sequelae.

**Figure 4:**
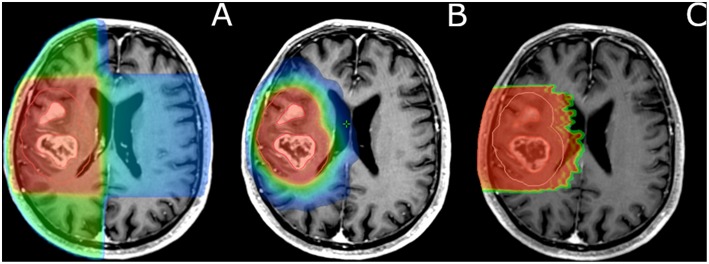
Comparison of dose distribution and target coverage (GBM) in 3D-CRT **(A)**, IMRT **(B)**, and protontherapy **(C)**. **(B,C)** show finer target coverage with increased normal tissue sparing. For clinical implementation of dose painting, these accurate RT techniques are needed **(B,C)**. In current routine clinical practice, the target volume receives a homogeneous dose prescription and distribution regardless of potential hypoxic subvolumes.

Several methods have been identified to overcome the dose-limiting tolerance of the brain, especially in the era of constant technological medical advancements. The improved resolution of MRIs allows better visualization of the brain anatomy and, in consequence, a more accurate delineation of organs at risk (OARs). Furthermore, newer RT planning techniques such as intensity-modulated radiation therapy (IMRT) make dose painting feasible. Compared to 3-D conformational radiotherapy (3D-CRT), IMRT allows highly conformal dose distributions of X-rays in target volumes with low levels of radiation to the surrounding normal tissues (74) (Figures 1–4). Using IMRT, very steep dose gradients in tumor subvolumes without unacceptable increased doses to OARs are achievable (53, 63, 64, 75).

Boron neutron capture therapy (BNCT) is another way to enhance the dose delivery in the tumor while preserving surrounding tissues. It can be done by boron administration into the tumor via the intravenous route or by perioperative intratumoral injection. BNCT relies on epithermal neutrons, which below 10 keV are not toxic to healthy tissues. Excellent spatial distribution is, however, critical due to their lack of spatial selectivity, with depth distribution profiles like photons but with a 3-fold biological efficacy, which can thus turn into a drawback if not targeted properly. Also, obtaining only a low energy spectrum of neutrons (below 10 keV to protect healthy tissues) can be quite challenging, and specific equipment has been designed that might only be adequate for superficial tumors (10 cm deep). Recent approaches suggest that proton and carbon ion beams could also be used to produce epithermal neutrons at the site of boron capture within the tumor (76–80). Thus, the need for specific neutron therapy machines, which are likely inadequate for the treatment of deep-seated tumors, might be surpassed by the use of proton and carbon ion accelerators. BCNT techniques are being investigated by a few teams worldwide, mostly in Japan and Sweden.

While being relevant from a radiobiological point of view, the concept of HIGRT has not entered clinical routine utilization, with some limiting factors being the difficulties tied to OER modeling, ptO_2_ mapping, and evolution of hypoxia during the course of RT.

## Reoxygenation Strategies to Improve RT Efficacy

### Reoxygenation During the Course of RT

The adaptation of RT based on hypoxia imaging also raises some questions about the evolution of hypoxia during the course of RT. Tumor reoxygenation is a phenomenon wherein cells that are hypoxic before RT become oxygenated during or after RT (81). For example, in head and neck cancers, it was recently published that during the course of RT, tumor hypoxia decreases (82). In this review, authors also discuss oxygenation in various tumor types, namely, lung, cervical, and rectal carcinomas. For these tumors, a decrease in hypoxia was likewise seen during RT. Thus, existing OER models do not incorporate variations of a tumor's radiosensitivity or reoxygenation during the course of treatment.

Rapid reoxygenation affects acutely hypoxic cells, while slow reoxygenation affects chronically hypoxic cells. These two processes may provide specific windows of opportunity. The RT fraction should be delivered when tumor reoxygenation is expected to be at its maximum so as to optimize the OER. Consequently, the HTV may not be spatially fixed over time, and a single pre-treatment PET may not be pertinent, especially for adaptive RT (83). PET scans may be repeated (over 5–7 days) to monitor hypoxia dynamics during RT (84). To this aim, numerous studies, mostly in head and neck cancers, have been published (82). Consistent with the reoxygenation model, results show that PET hypoxia uptake decreases during RT (82). Increasing PET uptake during RT has been correlated with loco-regional failure (85–87); however, disappearance of hypoxia was not correlated with better prognosis (88, 89).

With regard to the reproducibility of intratumor uptake among repeated scans during RT, results are ambiguous, with a study reporting highly reproducible uptake (90) and another reporting high uptake variability (51). Nevertheless, repeat imaging during the course of treatment might improve measurements (83). It is clear that further work is required to understand the spatio-temporal intratumor distribution of radiotracers before and during RT.

### External Reoxygenation Strategies

New radiosensitizing drugs and radio-enhancing nanoparticles may be delivered into the tumor to improve oxygenation. Among the radiosensitizers, some have been designed so as to overcome the effect of hypoxia by inducing reoxygenation of the tumor [reviewed in Graham and Unger (91)]. Of these, fluorochemicals can dissolve considerable amounts of oxygen and could be considered to deliver oxygen through passive diffusion in hypoxic regions. As an example, NVX-108 is a radiosensitizer composed of dodecafluoropentane (DDFP) exhibiting 200 times the oxygen carrying capacity compared to human hemoglobin (92), that demonstrated its promise in preclinical studies, with a clinical study ongoing for GBM.

Breathing of oxygen under normobaric or hyperbaric conditions has also been investigated. As discussed by Graham et al. hyperbaric oxygenation has an overall positive effect on RT but has not been adapted and remains to be validated as standard treatment. To further improve the reoxygenation, the use of carbogen has been proposed for GBM. However, overall results were unsatisfactory, and we recently demonstrated using advanced MRI that this failure was attributable to facilitated reoxygenation in the normal brain relative to the tumor (93).

In endogenous reoxygenation or external reoxygenation strategies, one can observe that hypoxia remains highly dynamic during the course of treatment. This reinforces the need for accurate imaging strategies that quantify temporal variations in tumor hypoxia to be able to adapt the RT regimen based on the hypoxic component of the tumor.

## Innovative Radiation Therapy Modalities to Overcome Hypoxia-Induced Radioresistance in GBM

The efficacy of photon-based RT critically depends on the presence of molecular oxygen. To achieve higher equivalent doses into the tumor, hadrontherapy such as proton therapy has also been proposed, advantageous due to its better spatial distribution and normal tissue sparing (and thus potential for accurate dose escalation). Carbon ion therapy is also a promising option, representing an increase in the biological efficacy of RT by a factor of 3 to 4 relative to photons, thus potentially overcoming radioresistance and achieving better tumor control while sparing healthy tissues.

### Proton Therapy

The depth dose distribution of a proton beam, represented by the Bragg peak, can be used to reduce radiation exposure of healthy tissues beyond the tumor (94) (Figure 4). These properties are particularly relevant to pediatric malignancies and benign/low-grade intracranial tumors. However, GBMs are rapidly progressive, poorly limited tumors. Thus, proton therapy should be used carefully to avoid marginal misses, with careful monitoring of tumor volumes over the weeks of RT. The process of rescanning, and replanning if necessary, is called adaptive RT. Provided that such caution is employed, proton therapy may be used to perform dose escalation. Proton therapy has a relative biological effectiveness (RBE) relative to high-energy photons of 1.1. Thus, protons are 10% more biologically efficient than high-energy photons. Although the OER of protons is similar to that of photons, the increased RBE might partially counteract the radioresistance of hypoxic areas. A dosimetric study indicated that for a subpopulation of patients with GBM, at least 90 Gray RBE (Gy RBE) could be delivered to the tumor with proton therapy, with only small volumes of normal brain structures receiving more than 70 Gy RBE. In a phase I–II proton therapy–based dose escalation study by Mizumoto et al. patients received photon-based RT or 250 MeV proton therapy (50.4 Gy RBE in 28 fractions) to a large tumor volume with a concomitant proton therapy boost (23.1 Gy RBE in 14 fractions) to MRI gadolinium-enhanced areas, which included hypoxic zones (95, 96). Overall, patients received a total dose of 96.6 Gy RBE in 56 fractions. The 1- and 2-year overall survival rates were 78% (95% CI, 61%−95%) and 43% (95% CI, 23%−63%), respectively, with a median survival of 21.0 months (range, 5.5–81.0 months; 95% CI, 16.1–25.9 months). This proof-of-concept study shows an overall survival gain of 6 months in comparison with historical series, but results have yet to be reproduced.

### Carbon Ion Beam Irradiation

Carbon ions have, to an even higher degree, the spatial selectivity of protons and can exhibit a very high LET of ~100 keV/μm. Carbon ions are densely ionizing, releasing their energy in a constant and very close manner, contrary to photons or protons. They possess, physical doses being equal, a higher RBE (around 3), as they more likely interact with DNA and produce complex damage that is difficult or impossible to repair (97). This direct effect of carbon ions is less influenced by the presence of oxygen. OER values of hypoxic cells are, respectively, 1.5 for high-LET ions and 3.0 for X-rays. For a similar effect in hypoxic conditions, the dose needed for conventional RT is three times higher than in normoxic conditions, but such increase in dose is not achievable without compromising OAR dose limits. For carbon ions, the 1.5 × increase needed is achievable. Preclinical studies have reported that accelerated heavy ion particles may have an advantage over X-rays in overcoming GBM radioresistance (98, 99). A phase I–II study combined 50 Gy X-ray RT with chemotherapy, followed by a carbon ion boost in the contrast enhancing region with doses from 16.8 to 24.8 Gray (RBE) (100). For the 32 GBM patients included, the median survival time was 17 months and reached 26 months for the high-dose group, with dose escalation having a significant impact. In line with these results, the randomized CLEOPATRA trial compares low- and high-LET irradiation in GBM patients (101).

### Spatial Fractionation, Hypofractionation, and Flash Dose

Alternative approaches also include modulation of radiation delivery to deliver tumoricidal doses to large volumes, using adaptations that allow an enhanced differential effect between normal tissues and the tumor. Spatial fractionation has been identified as a promising approach to such aim. This is particularly relevant to GBMs because of the large volumes irradiated and the radiosensitivity of the brain (102–104). Specific devices are being designed and adapted on various types of treatment machines using different radiation modalities, including synchrotron radiation, very-high-energy electrons, and proton beams (either double scattering with a grid or with modified pencil beam scanning).

Hypofractionation has been originally defined as the use of doses above 2.5 Gy per fraction. However, the concept of hypofractionation has now been extended to very high doses per fraction using photon-based stereotactic irradiation. Fraction doses commonly use 3 times 20 Gy (in lung cancers) but may even use 90 Gy in a single fraction for conditions such as trigeminal neuralgia. An extension of the concept is a flash (ultra-high) dose that combines hypofractionation with a very high dose rate (105, 106). Animal models have consistently shown excellent skin sparing and tumor response equivalent to standard regimens (107, 108).

## Conclusions

It is widely accepted that hypoxia is a poor prognostic factor in GBM. Among the key effects of hypoxia, radioresistance is a promising and potentially actionable factor. Imaging offers the opportunity to map tumor hypoxia or oxygenation before and during the course of RT and consequently opens an avenue for treatment adaptation. These adaptations can be by modulating doses based on ptO_2_ and OER measurements, by introducing reoxygenation strategies in combination with conventional RT, or by adapting the RT techniques. All these developments require accurate characterization of hypoxia. In this review, we argue that while various strategies are being developed, at present, PET remains the most relevant strategy with the most evidence.

## Author Contributions

All authors listed have made a substantial, direct and intellectual contribution to the work, and approved it for publication.

### Conflict of Interest Statement

The authors declare that the research was conducted in the absence of any commercial or financial relationships that could be construed as a potential conflict of interest.
